# Supplementary material for: Revealed versus potential spatial accessibility of healthcare and changing patterns during the COVID-19 pandemic
Source: Commun Med (Lond). 2023 Nov 3;3:157. doi: 10.1038/s43856-023-00384-9 (PMC10624905; doi:10.1038/s43856-023-00384-9)
Supplement: Supplementary file 1 — Supplementary Material [file 43856_2023_384_MOESM1_ESM.pdf]

Revealed versus potential spatial accessibility of healthcare and changing patterns during the COVID-19 pandemic

Kristina Gligorić[1,2*], Chaitanya Kamath[1*], Daniel J. Weiss[3,4*], Shailesh Bavadekar[1], Yun Liu[1], Tomer Shekel[1], Kevin Schulman[5**], Evgeniy Gabrilovich[1**†]

Affiliations
[1]Google Research, Mountain View, CA, USA
[2]Computer Science Department, Stanford University, Stanford, CA, USA
[3]Telethon Kids Institute, Perth Children's Hospital, Nedlands, WA, Australia
[4]Faculty of Health Sciences, Curtin University, Bentley, WA, Australia
[5]Clinical Excellence Research Center, School of Medicine and Graduate School of Business, Stanford University, Stanford, CA, USA

* These authors contributed equally.
** These authors jointly supervised this work.
† Corresponding author: egabrilovich@gmail.com

Supplementary Information

This supplementary material is organized as follows. In Section 1, we discuss the representativeness of location data. In Section 2, we provide supplementary details regarding how trips are constructed from raw location data. In Section 3, we provide the visualizations of revealed travel time to healthcare facilities by public transportation and walking. In Section 4 we describe supplementary analyses comparing the inventory of facilities used in this study with publicly available inventories. In Section 5 we discuss the global breakdown of the modes of transportation used to reach healthcare facilities. In Section 6 we provide a complete list of countries analyzed in this study. In Section 7, we describe supplementary analyses comparing revealed and potential accessibility in terms of distance traveled and time. Finally, in Section 8, we describe supplementary analyses regressing health outcomes on potential and revealed accessibility while controlling for wealth.

Supplementary Note 1. Supplementary discussion about the representativeness of location data

We first note that the representativeness of location data is difficult to analyze precisely because of the need to respect user privacy. However, pertinent aggregate insights are available from reports by the Pew Research Center[5] among others. Specifically regarding urbanization, smartphone ownership ranges from 80% to 89% across rural, suburban and urban U.S. adults, and with respect to income levels, rise from 76% for <$30K, to 96% for >$75K — indicating fairly high penetration at all income levels across both urban and rural areas. Though more of a hypothesis, we believe that the skew with respect to income or urban location is likely to over-represent urban areas that have relatively better access to healthcare facilities, and thus under-estimate the true revealed accessibility.

Globally, latest estimates of smartphone penetration are high at 83%,[8] and promisingly, there are signs that the “digital divide” with respect to age, such as smartphone ownership and other tech use is shrinking.[9] However, some skew is likely to remain in the foreseeable future, whether for historical data or newly collected data going forward. As noted in our manuscript, caution is warranted in interpretation of the results of the study.

Finally, regarding users with the Google location history feature enabled, a 2018 study surveyed more than 1000 users across 5 countries.[4] While many (20-50% of users) did not know their location history feature status, the vast majority of the remaining users had location history enabled (roughly 8:1 ratio of enabled:disabled for Japan, 3:1 for UK, 14:1 for Brazil, 5:1 for US, and 7:1 for Mexico). The authors concluded that (passive) location history data, when appropriately anonymized, was a novel and valuable way to study spatial movement while avoiding issues such as recall biases from self-report, or insufficient granularity from “CDR” (cell towers).

Supplementary Note 2. Supplementary discussion about trip definitions

Because GPS point logs are somewhat noisy, these points need to be processed to enable useful insights to be extracted. Our research concerns the notion of “trips”, which are travels from point A to B and need to exclude movements within the vicinity of A and within the vicinity of B, but incorporate temporary pauses (e.g., waiting at traffic lights whether walking, driving, or on public transportation). Briefly, the GPS point logs are first filtered to remove implausible points (e.g., “jitter” indicating unrealistic velocities). Next, a series of clustering algorithms (lead-based clustering, mean-shift clustering, and adaptive radius clustering) are used to distinguish dwell points from commute points. The commute points are used together with Google routing to define start and end points of trips. These post-processed trips are visible to the user via the “timeline” feature in the Google Maps app, and more technical details were described previously.[6] In this study, trips used were filtered for those originating from residential areas, following the categorization detailed previously.[7]

Supplementary Note 3. Revealed travel times visualizations for alternative modes of transport

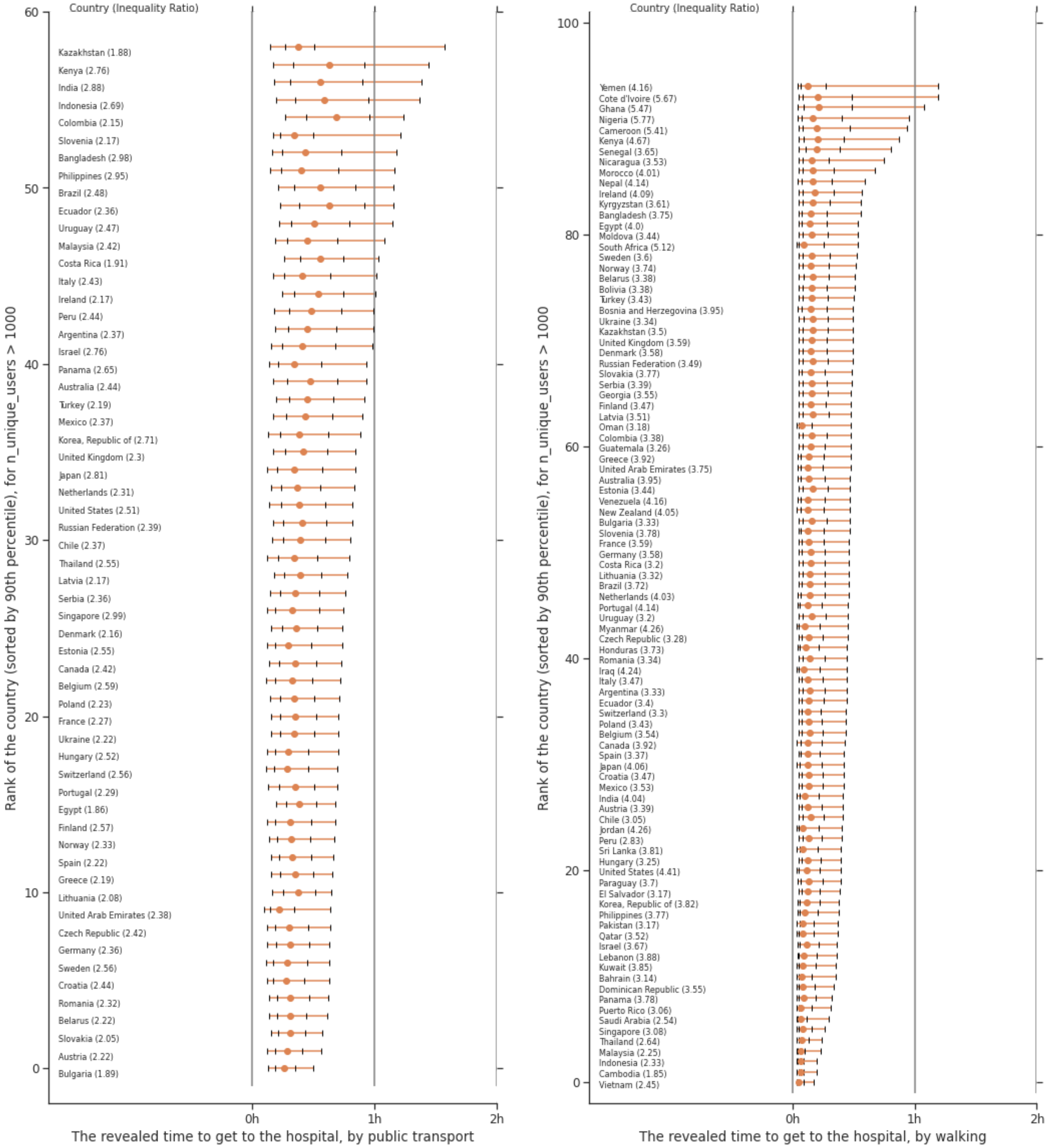

Supplementary Figure 1. Revealed travel time to healthcare facilities by public transportation (a) and walking (b). The inequality ratio is shown in brackets after the country name and illustrated in purple bars. Orange dots represent the median travel time in a country and vertical bars represent percentiles: 10th, 25th, 75th, and 90th. Countries are sorted by the 90th percentile.

Supplementary Note 4. Supplementary information about the inventory of healthcare providers

To assess both the coverage and the quality of our inventory of healthcare facilities (n≈214,000 facilities), we compare it with a global, publicly available set of hospitals and clinics (n≈157,000 facilities).[1] We allow a 500m radius around exact coordinates of facilities in our inventory to account for possible uncertainty of the coordinates. Globally, n≈82,000 facilities overlapped between our lists; in other words 53% of facilities that appear in the healthsites.io inventory also appear in our inventory and 38% of facilities that appear in our inventory are also part of the healthsites.io inventory. Establishing locations of healthcare facilities at global scale is known to be challenging.[2] The Jaccard coefficient between our inventory of facilities and healthsites.io was 0.29.[3]

A more detailed breakdown of these facilities counts and overlaps is presented below in Supplementary Table 1.

Income group (Categorized by World Bank)	Facility count		
	Our study	healthsites.io	Overlap
High income	86600	79144	45022
Upper middle income	54087	39809	21465
Lower middle income	72487	31335	15141
Low income	878	6089	673
Total	214052	156377	82301

Supplementary Table 1. Facility count stratified by income group.

Supplementary Note 5. Global breakdown of the different modes of transportation used to reach healthcare facilities

In this section, we provide additional information about the different modes of transportation analyzed in this paper. Supplementary Table 2 reports the global breakdown of the modes of transportation used to reach healthcare facilities, for the three main modes of transportation (passenger vehicle, public transportation, and walking).

Time period (year and quarter)	Passenger Vehicle (%)	Public transportation (%)	Walking (%)
2019_Q1	70.21	6.09	23.7
2019_Q2	70.15	5.37	24.48
2019_Q3	69.76	4.88	25.36
2019_Q4	72.32	4.4	23.28
2020_Q1	76.93	3.4	19.67
2020_Q2	77.96	2.17	19.87
2020_Q3	76.73	3.29	19.98
2020_Q4	77.51	3.17	19.32
2021_Q1	74.63	3.42	21.95
2021_Q2	73.63	3.55	22.82
2021_Q3	72.08	3.59	24.33

Supplementary Table 2. Global breakdown of the modes of transportation during the analysis period, per quarter.
Percentages are normalized per time period.

Supplementary Note 6. Studied countries.

A-C	D-I	J-N	O-S	T-Z
Afghanistan,*,**<br>Antigua and Barbuda,*,**<br>Argentina<br>Aruba,*,**<br>Australia<br>Austria<br>Bahrain,*<br>Bangladesh<br>Barbados,*,**<br>Belarus<br>Belgium<br>Benin,*,**<br>Bolivia,*<br>Bosnia and Herzegovina,*<br>Brazil<br>Bulgaria<br>Burkina Faso,*,**<br>Cambodia,*<br>Cameroon,*<br>Canada<br>Chile<br>Colombia<br>Costa Rica<br>Cote d'Ivoire,*<br>Croatia<br>Czech Republic	Denmark<br>Dominican Republic,*<br>Ecuador<br>Egypt<br>El Salvador,*<br>Estonia<br>Fiji,*,**<br>Finland<br>France<br>Gabon,*,**<br>Georgia,*<br>Germany<br>Ghana,*<br>Greece<br>Guatemala,*<br>Haiti,*,**<br>Honduras,*<br>Hungary<br>India<br>Indonesia<br>Iraq,*<br>Ireland<br>Israel<br>Italy	Jamaica,*,**<br>Japan<br>Jordan,*<br>Kazakhstan<br>Kenya<br>Kuwait,*<br>Kyrgyzstan,*<br>Latvia<br>Lebanon,*<br>Libya,*,**<br>Lithuania<br>Luxembourg,*<br>Malaysia<br>Mali,*,**<br>Malta,*,**<br>Mauritius,*,**<br>Mexico<br>Moldova,*<br>Morocco,*<br>Myanmar,*<br>Namibia,*,**<br>Nepal,*<br>Netherlands<br>New Zealand<br>Nicaragua,*<br>Niger,*,**<br>Nigeria,*<br>Norway	Oman,*<br>Pakistan,*<br>Panama<br>Paraguay,*<br>Peru<br>Philippines<br>Poland<br>Portugal<br>Puerto Rico,*<br>Qatar,*<br>Romania<br>Russia<br>Rwanda,*,**<br>Saudi Arabia,*<br>Senegal,*<br>Serbia<br>Singapore<br>Slovakia<br>Slovenia<br>South Africa,*<br>South Korea<br>Spain<br>Sri Lanka<br>Sweden<br>Switzerland	Tajikistan,*<br>Tanzania,*<br>Thailand<br>Togo,*,**<br>Trinidad and Tobago,*,**<br>Turkey<br>Uganda,*<br>Ukraine<br>United Arab Emirates<br>United Kingdom<br>United States<br>Uruguay<br>Venezuela,*<br>Vietnam<br>Yemen,*<br>Zambia,*,**<br>Zimbabwe,*,**

Supplementary Table 3. Studied countries/regions for revealed accessibility.
* indicates public transit data not available; ** indicates walking data not available.

A-B	C-E	F-J	K-M	N-R	S-S	T-Z
Afghanistan*<br>Aland Islands<br>Albania*<br>Algeria*<br>Andorra*<br>Angola*<br>Anguilla*<br>Antigua and Barbuda*<br>Argentina<br>Armenia<br>Aruba*<br>Australia<br>Austria<br>Azerbaijan<br>Bahrain<br>Bangladesh<br>Barbados*<br>Belarus<br>Belgium<br>Belize*<br>Benin*<br>Bermuda*<br>Bhutan*<br>Bolivia*<br>Bosnia and Herzegovina<br>Botswana*<br>Brazil<br>Brunei Darussalam*<br>Bulgaria<br>Burkina Faso*<br>Burundi*	Cambodia*<br>Cameroon*<br>Canada<br>Cape Verde*<br>Central African Republic*<br>Chad*<br>Chile<br>Colombia<br>Comoros*<br>Costa Rica<br>Cote d'Ivoire*<br>Croatia<br>Cuba*<br>Cyprus*<br>Czech Republic<br>Democratic Republic of Congo*<br>Denmark<br>Djibouti*<br>Dominican Republic<br>Ecuador<br>Egypt<br>El Salvador*<br>Equatorial Guinea*<br>Eritrea*<br>Estonia<br>Ethiopia*	Fiji*<br>Finland<br>France<br>French Guiana<br>French Polynesia*<br>Gabon*<br>Gambia*<br>Georgia<br>Germany<br>Ghana*<br>Gibraltar<br>Greece<br>Greenland*<br>Guadeloupe*<br>Guatemala*<br>Guernsey*<br>Guinea*<br>Guinea-Bissau *<br>Guyana*<br>Haiti*<br>Honduras*<br>Hungary<br>Iceland<br>India<br>Indonesia<br>Iran*<br>Iraq*<br>Ireland<br>Israel<br>Italy<br>Jamaica<br>Jordan*	Kazakhstan<br>Kenya<br>Kiribati*<br>Kuwait<br>Kyrgyzstan*<br>Laos*<br>Latvia<br>Lebanon<br>Lesotho*<br>Liberia*<br>Libya*<br>Liechtenstein<br>Lithuania<br>Luxembourg<br>Macao<br>Macedonia<br>Madagascar*<br>Malawi*<br>Malaysia<br>Maldives*<br>Mali*<br>Malta<br>Martinique*<br>Mauritania*<br>Mauritius*<br>Mayotte*<br>Mexico<br>Moldova<br>Mongolia<br>Montenegro*<br>Montserrat*<br>Morocco<br>Mozambique*<br>Myanmar*	Nepal*<br>Netherlands<br>New Caledonia*<br>New Zealand<br>Nicaragua*<br>Niger*<br>Nigeria<br>Norfolk Island*<br>Norway<br>Oman<br>Pakistan<br>Panama<br>Papua New Guinea*<br>Paraguay<br>Peru<br>Philippines<br>Poland<br>Portugal<br>Puerto Rico<br>Qatar<br>Republic of Congo*<br>Romania<br>Russian Federation<br>Rwanda*	Saint Kitts and Nevis*<br>Saint Lucia*<br>Saint Martin (French part)*<br>Saint Pierre and Miquelon*<br>Sao Tome and Principe*<br>Saudi Arabia*<br>Senegal*<br>Serbia<br>Sierra Leone*<br>Singapore<br>Slovakia<br>Slovenia<br>Somalia*<br>South Africa<br>South Sudan*<br>Spain<br>Sri Lanka<br>Sudan*<br>Suriname*<br>Swaziland*<br>Sweden<br>Switzerland	Tajikistan*<br>Tanzania*<br>Thailand<br>Timor-Leste*<br>Togo*<br>Trinidad and Tobago*<br>Tunisia<br>Turkey<br>Turkmenistan*<br>Tuvalu*<br>Uganda*<br>Ukraine<br>United Arab Emirates<br>United Kingdom<br>United States<br>Uruguay<br>Uzbekistan<br>Vanuatu*<br>Venezuela*<br>Vietnam<br>Virgin Islands*<br>Yemen*<br>Zambia<br>Zimbabwe*

Supplementary Table 4. Studied countries/regions for potential accessibility.
* indicates public transit data not available

Supplementary Note 7. Comparing revealed and potential accessibility.

a

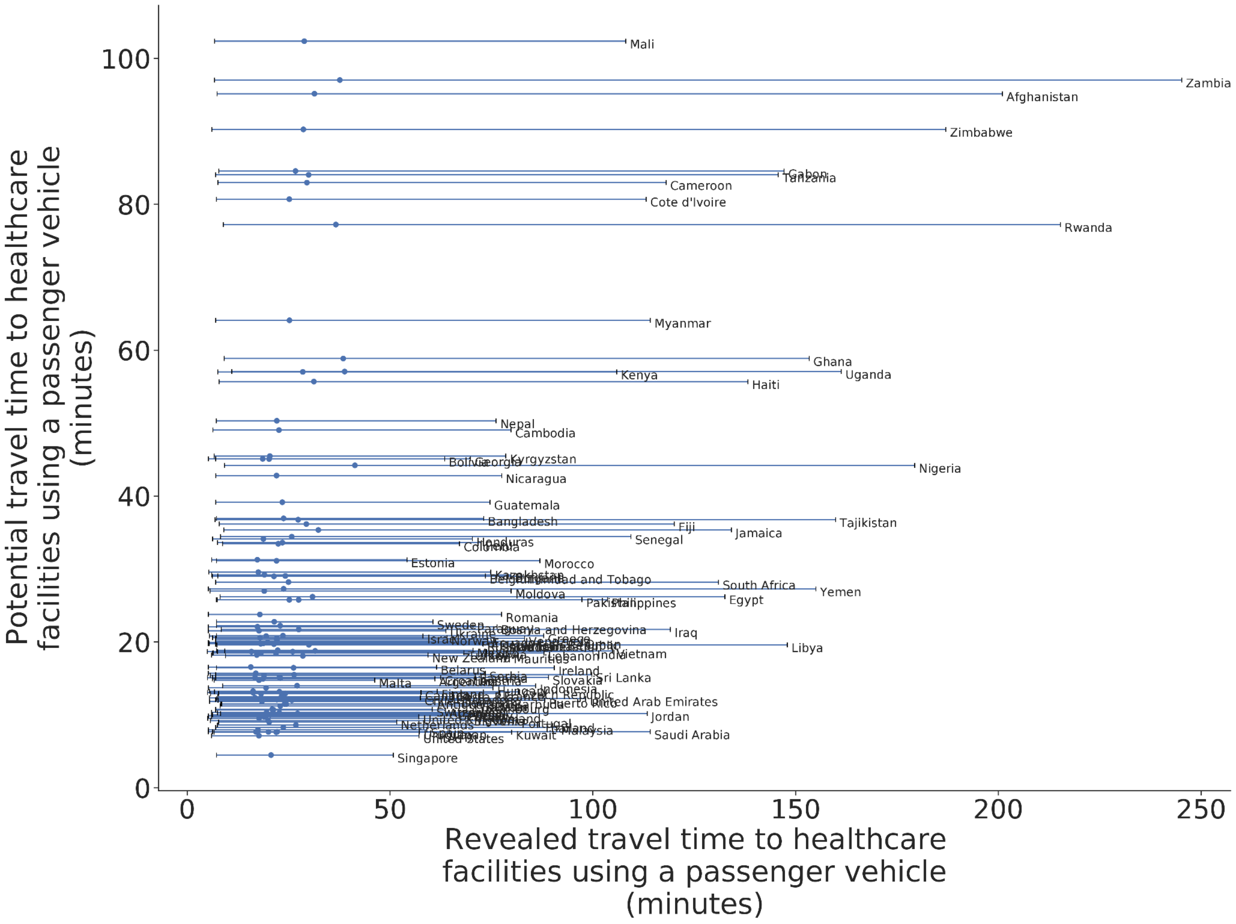

b

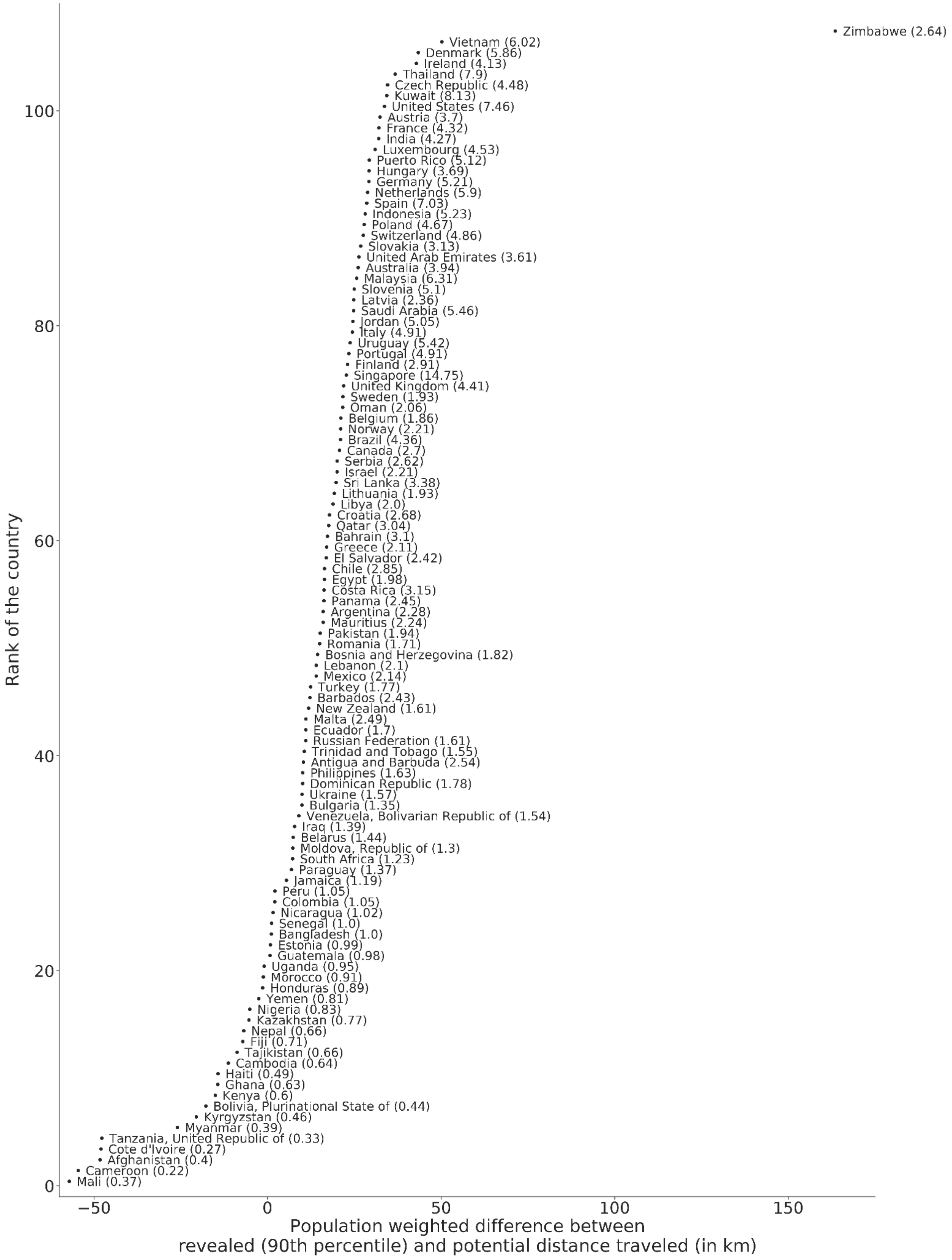
• Zimbabwe (2.64)
• Vietnam (6.02)
• Denmark (5.86)
• Ireland (4.13)
• Thailand (7.9)
• Czech Republic (4.48)
• Kuwait (8.13)
• United States (7.46)
• Austria (3.7)
• France (4.32)
• India (4.27)
• Luxembourg (4.53)
• Puerto Rico (5.12)
• Hungary (3.69)
• Germany (5.21)
• Netherlands (5.9)
• Spain (7.03)
• Indonesia (5.23)
• Poland (4.67)
• Switzerland (4.86)
• Slovakia (3.13)
• United Arab Emirates (3.61)
• Australia (3.94)
• Malaysia (6.31)
• Slovenia (5.1)
• Latvia (2.36)
• Saudi Arabia (5.46)
• Jordan (5.05)
• Italy (4.91)
• Uruguay (5.42)
• Portugal (4.91)
• Finland (2.91)
• Singapore (14.75)
• United Kingdom (4.41)
• Sweden (1.93)
• Oman (2.06)
• Belgium (1.86)
• Norway (2.21)
• Brazil (4.36)
• Canada (2.7)
• Serbia (2.62)
• Israel (2.21)
• Sri Lanka (3.38)
• Lithuania (1.93)
• Libya (2.0)
• Croatia (2.68)
• Qatar (3.04)
• Bahrain (3.1)
• Greece (2.11)
• El Salvador (2.42)
• Chile (2.85)
• Egypt (1.98)
• Costa Rica (3.15)
• Panama (2.45)
• Argentina (2.28)
• Mauritius (2.24)
• Pakistan (1.94)
• Romania (1.71)
• Bosnia and Herzegovina (1.82)
• Lebanon (2.1)
• Mexico (2.14)
• Turkey (1.77)
• Barbados (2.43)
• New Zealand (1.61)
• Malta (2.49)
• Ecuador (1.7)
• Russian Federation (1.61)
• Trinidad and Tobago (1.55)
• Antigua and Barbuda (2.54)
• Philippines (1.63)
• Dominican Republic (1.78)
• Ukraine (1.57)
• Bulgaria (1.35)
• Venezuela, Bolivarian Republic of (1.54)
• Iraq (1.39)
• Belarus (1.44)
• Moldova, Republic of (1.3)
• South Africa (1.23)
• Paraguay (1.37)
• Jamaica (1.19)
• Peru (1.05)
• Colombia (1.05)
• Nicaragua (1.02)
• Senegal (1.0)
• Bangladesh (1.0)
• Estonia (0.99)
• Guatemala (0.98)
• Uganda (0.95)
• Morocco (0.91)
• Honduras (0.89)
• Yemen (0.81)
• Nigeria (0.83)
• Kazakhstan (0.77)
• Nepal (0.66)
• Fiji (0.71)
• Tajikistan (0.66)
• Cambodia (0.64)
• Haiti (0.49)
• Ghana (0.63)
• Kenya (0.6)
• Bolivia, Plurinational State of (0.44)
• Kyrgyzstan (0.46)
• Myanmar (0.39)
• Tanzania, United Republic of (0.33)
• Cote d'Ivoire (0.27)
• Afghanistan (0.4)
• Cameroon (0.22)
• Mali (0.37)
Rank of the country
100
80
60
40
20
0
−50
0
50
100
150
Population weighted difference between
revealed (90th percentile) and potential distance traveled (in km)

Supplementary Figure 2. Comparisons of potential and revealed access metrics.
(a) Scatter plot of potential versus reveal travel times by passenger vehicle. Dots and limits of the horizontal lines represent median and 10th/90th percentiles of potential travel times. (b) Comparison of revealed and potential distance traveled (distance analog of Figure 6).

Supplementary Note 8. Multivariable analysis for health outcomes.

Target variable	Independent variable	Coefficient (95%CI)	p
Life expectancy	Revealed travel time	-0.3248 (-0.467, -0.183)	0.000
	Potential travel time	-0.3677 (-0.512, -0.224)	0.000
	GDP (2017)	0.3278 (0.221, 0.435)	0.000
Infant mortality	Revealed travel time	0.3253 (0.160, 0.491)	0.000
	Potential travel time	0.4204 (0.252, 0.589)	0.000
	GDP (2017)	-0.2039 (-0.329, -0.079)	0.002
Life expectancy	Inequality ratio of revealed travel time	-0.5617 (-0.724, -0.400)	0.000
	Gini coefficient	-0.1857 (-0.348, -0.024)	0.025

Supplementary Table 5. Comparison between health outcomes and revealed travel times while controlling for potential travel time and GDP.

Supplementary References

1 Saameli R, Kalubi D, Herringer M, Sutton T, de Roodenbeke E. Healthsites.io: The Global Healthsites Mapping Project. Technologies for Development. 2018; : 53–9.

2 Weiss DJ, Nelson A, Vargas-Ruiz CA, *et al.* Global maps of travel time to healthcare facilities. *Nat Med* 2020; **26**: 1835–8.

3 Jaccard P. THE DISTRIBUTION OF THE FLORA IN THE ALPINE ZONE.1. New Phytologist. 1912; **11**: 37–50.

4 Ruktanonchai, N. W., Ruktanonchai, C. W., Floyd, J. R. & Tatem, A. J. Using Google Location History data to quantify fine-scale human mobility. *Int. J. Health Geogr.* 17, 28 (2018).

5 Demographics of Mobile Device Ownership and Adoption in the United States. Pew Research Center: Internet, Science & Tech. 2021; published online April 7. https://www.pewresearch.org/internet/fact-sheet/mobile/ (accessed March 25, 2022).

6 Kirmse, A., Udeshi, T., Bellver, P. & Shuma, J. Extracting patterns from location history. in *Proceedings of the 19th ACM SIGSPATIAL International Conference on Advances in Geographic Information Systems* 397–400 (Association for Computing Machinery, 2011).

7 Aktay, A. *et al.* Google COVID-19 Community Mobility Reports: Anonymization Process Description (version 1.0). *arXiv [cs.CR]* (2020).

8 HOW MANY SMARTPHONES ARE IN THE WORLD? Bankmycell. https://www.bankmycell.com/blog/how-many-phones-are-in-the-world (accessed Nov 22, 2022)

9 Faverio, M. Share of those 65 and older who are tech users has grown in the past decade. *Pew Research Center* https://www.pewresearch.org/fact-tank/2022/01/13/share-of-those-65-and-older-who-are-tech-users-has-grown-in-the-past-decade/ (2022) (accessed Nov 22, 2022)
