# Supplementary material for: Revealed versus potential spatial accessibility of healthcare and changing patterns during the COVID-19 pandemic
Source: Commun Med (Lond). 2023 Nov 3;3:157. doi: 10.1038/s43856-023-00384-9 (PMC10624905; doi:10.1038/s43856-023-00384-9)
Supplement: Supplementary file 2 — Reporting Summary [file 43856_2023_384_MOESM2_ESM.pdf]

nature portfolio

Corresponding author(s): Evgeniy Gabrilovich

Last updated by author(s): Aug 25, 2023

Reporting Summary

Nature Portfolio wishes to improve the reproducibility of the work that we publish. This form provides structure for consistency and transparency in reporting. For further information on Nature Portfolio policies, see our Editorial Policies and the Editorial Policy Checklist.

Statistics

For all statistical analyses, confirm that the following items are present in the figure legend, table legend, main text, or Methods section.

n/a	Confirmed	
☐	☒	The exact sample size (*n*) for each experimental group/condition, given as a discrete number and unit of measurement
☐	☒	A statement on whether measurements were taken from distinct samples or whether the same sample was measured repeatedly
☐	☒	The statistical test(s) used AND whether they are one- or two-sided<br>*Only common tests should be described solely by name; describe more complex techniques in the Methods section.*
☐	☒	A description of all covariates tested
☐	☒	A description of any assumptions or corrections, such as tests of normality and adjustment for multiple comparisons
☐	☒	A full description of the statistical parameters including central tendency (e.g. means) or other basic estimates (e.g. regression coefficient) AND variation (e.g. standard deviation) or associated estimates of uncertainty (e.g. confidence intervals)
☐	☒	For null hypothesis testing, the test statistic (e.g. *F*, *t*, *r*) with confidence intervals, effect sizes, degrees of freedom and *P* value noted<br>*Give P values as exact values whenever suitable.*
☒	☐	For Bayesian analysis, information on the choice of priors and Markov chain Monte Carlo settings
☒	☐	For hierarchical and complex designs, identification of the appropriate level for tests and full reporting of outcomes
☐	☒	Estimates of effect sizes (e.g. Cohen's *d*, Pearson's *r*), indicating how they were calculated

Our web collection on statistics for biologists contains articles on many of the points above.

Software and code

Policy information about availability of computer code

Data collection	The inventory of geolocated hospitals and medical centers providing urgent and emergency care was extracted from Google's Maps and Search datasets. Revealed accessibility is computed using aggregated, anonymized data from users who have turned on the Location History setting, which is off by default. People who have Location History turned on can choose to turn it off at any time from their Google Account, and can always delete Location History data directly from their Timeline. Potential travel times were estimated using Google Maps driving directions. The anonymized data and code needed to reproduce the analyses are provided as Supplementary Data 1.
Data analysis	Data analysis was performed using python and open-source libraries (pandas, matplotlib, scipy).

For manuscripts utilizing custom algorithms or software that are central to the research but not yet described in published literature, software must be made available to editors and reviewers. We strongly encourage code deposition in a community repository (e.g. GitHub). See the Nature Portfolio guidelines for submitting code & software for further information.

Data

Policy information about availability of data

All manuscripts must include a data availability statement. This statement should provide the following information, where applicable:

- Accession codes, unique identifiers, or web links for publicly available datasets
- A description of any restrictions on data availability
- For clinical datasets or third party data, please ensure that the statement adheres to our policy

The following aggregated and anonymized data reported throughout this manuscript is made available as part of Supplementary Data 1: revealed accessibility i.e. distribution of times (by quantiles) to reach the nearest medical facility, by country and mode of transportation (as reported in Figure 1); COVID-19 analysis (by

quarter over 2020-2021) i.e. percentage change in median travel time and median distance traveled, compared to the pre-pandemic baseline, reported globally (Figure 2) and pre-pandemic median travel time and absolute change therein during the pandemic, by country and mode of transportation (Figure 3); country-specific inequality measures, and correlation coefficients between the measures of revealed accessibility and healthcare outcomes (Figure 4); potential accessibility i.e. expected time in minutes to get to the nearest medical facility, by country and mode of transportation (Figure 5); and population-weighted difference between the revealed and potential travel time (in minutes), per country (Figure 6).

Field-specific reporting

Please select the one below that is the best fit for your research. If you are not sure, read the appropriate sections before making your selection.

☐ Life sciences ☒ Behavioural & social sciences ☐ Ecological, evolutionary & environmental sciences

For a reference copy of the document with all sections, see nature.com/documents/nr-reporting-summary-flat.pdf

Behavioural & social sciences study design

All studies must disclose on these points even when the disclosure is negative.

Study description	The study utilizes quantitative methods.
Research sample	The sample is not representative of the general population. Specifically, those who own and use GPS-equipped mobile phones and opt into location sharing are a representative sample of the general population. Evidence suggests that, particularly in developing countries, phone ownership skews towards wealthier individuals, younger ages, and males.
Sampling strategy	We do not sample users. We bound the contribution a single user can make to the distribution of travel times per geographic region, time period, and mode of transportation, to 1 by sampling a single contribution uniformly at random.
Data collection	See Data Collection and Sampling above.
Timing	The revealed accessibility dataset was collected between October and November 2021. Potential travel time estimates were estimated in August 2019 for the presented analyses, and again in July 2021 for robustness.
Data exclusions	We provide the list of analyzed countries in Figure 1 and Supplementary Table 3. Within the analyzed countries, there was no exclusion.
Non-participation	Does not apply. We do not sample users and all opt-in users contributed to the anonymized data.
Randomization	Does not apply. Participants are not assigned to groups.

Reporting for specific materials, systems and methods

We require information from authors about some types of materials, experimental systems and methods used in many studies. Here, indicate whether each material, system or method listed is relevant to your study. If you are not sure if a list item applies to your research, read the appropriate section before selecting a response.

Materials & experimental systems

n/a	Involved in the study	
☒	☐	Antibodies
☒	☐	Eukaryotic cell lines
☒	☐	Palaeontology and archaeology
☒	☐	Animals and other organisms
☒	☐	Human research participants
☒	☐	Clinical data
☒	☐	Dual use research of concern

Methods

n/a	Involved in the study	
☒	☐	ChIP-seq
☒	☐	Flow cytometry
☒	☐	MRI-based neuroimaging
