# Supplementary material for: Revealed versus potential spatial accessibility of healthcare and changing patterns during the COVID-19 pandemic
Source: Commun Med (Lond). 2023 Nov 3;3:157. doi: 10.1038/s43856-023-00384-9 (PMC10624905; doi:10.1038/s43856-023-00384-9)
Supplement: Supplementary file 4 — Description of Additional Supplementary Files [file 43856_2023_384_MOESM4_ESM.pdf]

Description of Additional Supplementary Files

File name: Supplementary Data 1
Description: A zip file containing all data (potential travel times and revealed travel times) and code needed to reproduce the analysis presented in the manuscript.
